# 1435. Incidence of Tuberculosis Infection Among New Prisoners in Southern Thailand

**DOI:** 10.1093/ofid/ofac492.1264

**Published:** 2022-12-15

**Authors:** Sujinda Ruangchan, Nongnuch Kiamkan, Sumonta Kabinlapat, Nichareekorn Jeduman, Aunjai Kasa, Khachornsakdi Silpapojakul

**Affiliations:** Songkhla Provincial Hospital, Songkhla Province, Songkhla, Thailand; Songkhla Provincial Hospital, Songkhla Province, Songkhla, Thailand; Songkhla Provincial Hospital, Songkhla Province, Songkhla, Thailand; Songkhla Provincial Hospital, Songkhla Province, Songkhla, Thailand; Songkhla Provincial Priosn, Songkhla, Songkhla, Thailand; Prince of Songkhla university, Songkha, Songkhla, Thailand

## Abstract

**Background:**

Tuberculosis in prisons was reported to be 100 times higher than the normal population. Late diagnosis, overcrowding, and poor ventilation encourage the transmission of tuberculosis. Five percent of new tuberculosis infections turn into active disease within two years. This study was conducted to determine the incidence of tuberculosis infection among new prisoners in southern Thailand.

**Methods:**

A prospective cohort study was planned for January 2020‒December 2021 at Songkhla Provincial Prison. However, due to the emerging COVID-19 pandemic, the study was terminated early in February 2021. All new prisoners aged ≥15 years were included. Subjects were excluded if they had any history of a previous diagnosis of tuberculosis, household contact with a tuberculosis patient, or a history of repeated imprisonment. Chest radiography together with a 2-step tuberculin skin test (TST) was done within 2 weeks after imprisonment and at 6 months using the one-step TST. A positive TST was defined by a skin reaction ≥1.5 cm. Prisoners with negative TST had a repeat TST at 6 months. New tuberculosis infection was defined by conversion of TST to positive after 6 months of imprisonment without evidence of active tuberculosis from chest radiography.

**Results:**

The number of new prisoners during the study period was 602. All prisoners were men with an average age of 31.11 (range 18‒75) years Fifty-one prisoners were excluded. Three hundred and eighty-five prisoners completed the 2-step TST. A total of 11.07% (61/551) of subjects had initially tested positive. Three hundred and seventy prisoners were followed. The COVID-19 pandemic caused a total shutdown of the study because access to the prison was blocked. Therefore, only 53 (9.6%) prisoners completed the study protocol at 6 months. Five prisoners (9.4%) were tuberculin converters with no evidence of active tuberculosis from chest radiography

**Figure:**
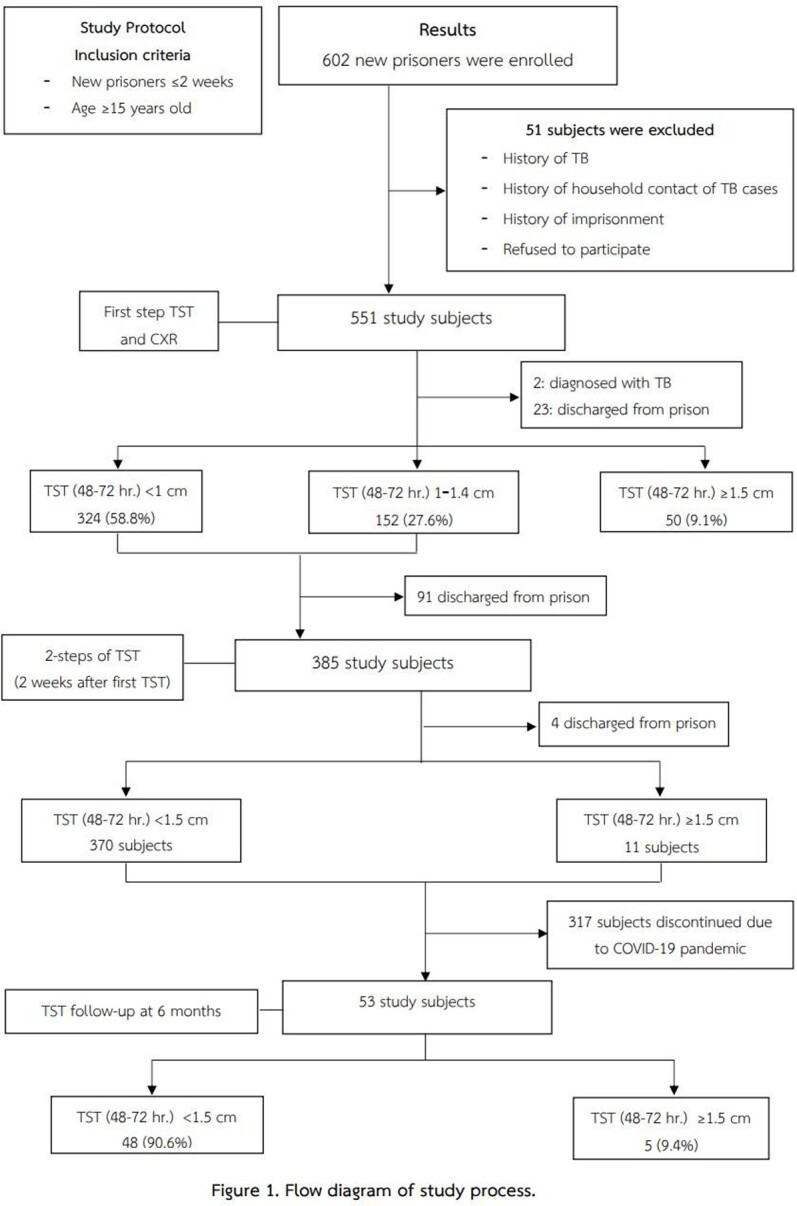

**Conclusion:**

The incidence of tuberculosis infection in new prisoners was 9.4%. This study has limitations. Due to the emerging COVID-19 pandemic, 90.4% of subjects were lost to follow-up and some prisoners were released before 6 months, which caused low power of the study. Further studies are needed to identify the incidence of new tuberculosis infection in new prisoners.

**Disclosures:**

**All Authors**: No reported disclosures.

